# Exploring the influence of short-term temperature patterns on temperature-related mortality: a case-study of Melbourne, Australia

**DOI:** 10.1186/s12940-016-0193-1

**Published:** 2016-11-10

**Authors:** John L. Pearce, Madison Hyer, Rob J. Hyndman, Margaret Loughnan, Martine Dennekamp, Neville Nicholls

**Affiliations:** 1 Department of Public Health Sciences, Medical University of South Carolina, 135 Cannon Street, Charleston, SC 29403 USA; 2 School of Geography and Environmental Science, Monash University, Wellington Rd., Clayton, Victoria 3800 Australia; 3 Department of Econometrics and Business Statistics, Monash University, Wellington Rd., Clayton, Victoria 3800 Australia; 4 Department of Epidemiology and Preventative Medicine, Monash University, 99 Commercial Rd., Melbourne, Victoria 3004 Australia

**Keywords:** Climate, Health, Heat events, Heat wave, Temperature-mortality, Weather

## Abstract

**Background:**

Several studies have identified the association between ambient temperature and mortality; however, several features of temperature behavior and their impacts on health remain unresolved.

We obtain daily counts of nonaccidental all-cause mortality data in the elderly (65 + years) and corresponding meteorological data for Melbourne, Australia during 1999 to 2006. We then characterize the temporal behavior of ambient temperature development by quantifying the rates of temperature change during periods designated by pre-specified windows ranging from 1 to 30 days. Finally, we evaluate if the association between same day temperature and mortality in the framework of a Poisson regression and include our temperature trajectory variables in order to assess if associations were modified by the nature of how the given daily temperature had evolved.

**Results:**

We found a positive significant association between short-term mortality risk and daily average temperature as mortality risk increased 6 % on days when temperatures were above the 90th percentile as compared to days in the referent 25–75th. In addition, we found that mortality risk associated with daily temperature varied by the nature of the temperature trajectory over the preceding twelve days and that peaks in mortality occurred during periods of high temperatures and stable trajectories and during periods of increasing higher temperatures and increasing trajectories.

**Conclusion:**

Our method presents a promising tool for improving understanding of complex temperature health associations. These findings suggest that the nature of sub-monthly temperature variability plays a role in the acute impacts of temperature on mortality; however, further studies are suggested.

## Background

It is well known that thermal stress is a major contributor in weather-related health burdens as epidemiologic studies of temperature exposure have well-illustrated that temperature influences population-level health in a nonlinear way, with extremes (hot or cold) tending to have the largest effects [1, 2]. This exposure-response relationship is complex as ‘hot’ and ‘cold’ effects reveal a ‘U’ or ‘J’ shaped dose–response with variable temporal patterns of association, as excessive heat typically demonstrates a rapid effect on mortality (less than 2 days) and the effects of excessive cold tend to be evident over a longer period (sometimes greater than two weeks) [1]. These effects have also been shown to persist or amplify over periods of successive days, a pattern often described as a heat or cold wave [3, 4]. Although findings have been consistent, the complex nature of this environmental health problem has led to many aspects remaining unresolved [5, 6].

One area of particular interest as of late has been temperature variability, as a changing climate is expected to not only increase average temperatures and the frequency of extreme temperature events but also the variability of temperature within seasons [7]. Recent research suggests that such concerns are warranted as temperature variability (i.e., swings in temperature) has been shown to be an important determinant of health [8–10]. For example, findings from an examination of within-day temperature variability (difference in daily min/max, a.k.a. diurnal temperature range) and day-to-day mean temperature differences in Brisbane, Australia suggest that temperature variability is associated with an increase in childhood pneumonia cases [9]. In east Asia, and examination of diurnal temperature range and mortality found greater effects on respiratory mortality and the elderly [11]. In the US, recent findings from an investigation focused on whether the standard deviation of summer temperatures was associated with survival in four cohorts of persons over age 65 years with predisposing diseases found that long-term increases in temperature variability may increase risk of mortality in certain populations [10]. Biologically speaking, such findings are plausible as it is well known that certain populations (e.g., elderly) have a more difficulty with thermoregulation and acclimatization, processes that may be challenged during periods of significant temperature shifts [12].

Collectively, these findings provide evidence that supports a hypothesis that changes in temperature can be harmful to health; however, more studies are needed to better understand how short-term variability in temperature behavior influences health. For example, it is still largely unclear whether or not rapid increases or decreases in temperatures influence health or if they act in combination with extremes to amplify effects. Such knowledge gaps are difficult to address and warrant the development of new methodologies.

In this study, our overarching objective is to illustrate a method that allows health investigators to explore the role of sub-monthly patterns of temperature change in the short-term relationship between temperature and human health. The driving hypothesis is that heterogeneity in the nature of temperature change over sub-monthly periods (i.e., temperature trajectory) will impact the magnitude of short-term temperature associations with our health outcome. To address this hypothesis, we apply our method within the framework of an acute health effects study of temperature and elderly mortality using Melbourne, Australia as our case study. The city of Melbourne, with a population of approximately 3.9 million, presents an appropriate study region because of its distinguishing temperature extremes, relatively large elderly population (13 % > 65 years in 2006), and established literature on heat-related mortality [4].

## Methods

This study is conducted in two stages. First, we develop a metric that characterizes the temporal trajectory of ambient temperature behavior by constructing linear models that define the pattern of temperature change (i.e., slope) over pre-specified windows of time. Then, we apply our metric in the framework of a well-established epidemiological modeling approach in order to estimate associations between temperature progressions and their interactions with ambient temperature on mortality while controlling for long-term trends and season.

### Data

The data used in this study are concurrent time-series of daily mortality counts and weather summaries from Melbourne, Australia over the years 1999 to 2006. Daily mortality data were provided by the Australian Bureau of Statistics (http://www.abs.gov.au/) and are the aggregate counts of non-accidental daily deaths of individuals aged 65 years and over (65+) across Greater Melbourne. Daily automatic weather station observations for air temperature (°C) and dew-point temperature (°C) were provided by the Bureau of Meteorology (www.bom.gov.au) for site number 086282 (Melbourne International Airport).

### Characterizing temperature trajectories

Conceptually, we define the ‘temperature trajectory’ of a daily temperature as the rate of change of temperature over the days preceding (pre-specified window) the current observation. For example, if today’s temperature is 28 °C and our window of interest is the preceding three days, then the trajectory is defined as the slope of temporal changes in temperature observations over those days. So, a positive slope indicates there was an overall trend in temperatures rising over the window of interest, a negative slope implies a decreasing trend, and a zero slope indicates stability or neither a decreasing or increasing trend.

We model temperature trajectories using ordinary least-squares regression, applied to windows of length *w*. Specifically, we define a given day’s temperature trajectory as the linear trend of temperature over the preceding *w* days. Let *T* be the total length of the time series of temperatures. Then for each value of *t* between *w* + 1 and *T*, we estimate a simple regression trend equation over the *w* observations prior to time *t*:

$$ {y}_i={\alpha}_{t,w}-{\beta}_{t,w}\left(t-i\right)+{\varepsilon}_{i,t}, $$

where *y*_*i*_ is the observed average temperature on day *i*, *ε*_*i,t*_ is a random error term, and *i* = *t* − *w*, …, *t*. Thus, *β*_*t*,*w*_ is the slope of the temperature trajectory for day *t* estimated from the days *t* − *w* through *t*. We consider window sizes *w* ranging from 1 to 30. Temperature windows were restricted to a 30 day maximum in order to focus on sub-monthly behaviors. For each trajectory, we evaluate basic statistical properties and relationships with daily temperature.

### Epidemiologic analyses

We modeled associations between temperature and daily mortality counts using the framework of a Poisson generalized linear model (GLM) allowing for overdispersion [13]. The dependent variable was the daily number of deaths in the elderly and the primary exposure of interest was ambient temperature. To control for potential confounding, our model included a natural spline term accounting for long-term trend and seasonality (degrees of freedom (df = 7 per year), an indicator term for day-of-the-week, a term for influenza hospitalizations (indicator of flu season), and a natural spline term for dew-point temperature, a measure of atmospheric moisture (df = 4). Using this base model, we estimate main effects for ambient temperature using same day average temperature and temperature trajectory using our previously described variable *β*_*t*,*w*_. Potential effect modification of temperature was explored using a product-term model that included terms for all variables and products contained within product.

The main effects of temperature and temperature trajectory were estimated by fitting a Poisson GLM model to the daily mortality counts with log mean given by (Model 1):

$$ \log \left({\mu}_t\right)=\alpha +{s}_1(t)+\mathrm{DO}{\mathrm{W}}_t+\delta \mathrm{F}\mathrm{L}{\mathrm{U}}_t+{s}_2\left(\mathrm{D}\mathrm{P}{\mathrm{T}}_t\right)+{s}_3\left({y}_t\right)+{s}_4\left({\beta}_{t,w}\right), $$

where *t* is the day in the study period, DOW_*t*_ is a day-of-week factor, FLU_*t*_ is the number of influenza-related hospital admissions, DPT_*t*_ is the daily mean dew-point temperature, and *s*_1_, …, *s*_4_ are all smooth functions estimated using natural splines.

Model 2 is identical to model 1 except that percentile-based categories were used instead of natural spline terms for temperatures and temperature trajectories. This can be expressed as:

$$ \log \left({\mu}_t\right)=\alpha +{s}_1(t)+\mathrm{DO}{\mathrm{W}}_t+\delta \mathrm{F}\mathrm{L}{\mathrm{U}}_t+{s}_2\left(\mathrm{D}\mathrm{P}{\mathrm{T}}_t\right)+{C}_t+{D}_t, $$

where *C*_*t*_ is a percentile-based category factor for temperature *y*_*t*_ and *D*_*t*_ is a percentile-based category factor for trajectory *β*_*t*,*w*_.

The effect modification of temperature with mortality was estimated with Model 3 by adding the product term *s*_5_(*y*_*t*_ × *β*_*t*,*w*_) to Model 1, where *s*_5_ is a smooth function estimated using natural splines. Models were fitted in the R statistical environment version 3.2.1 [14] using the glm() modeling function.

### Sensitivity analysis

As determination of the trajectory window length is an important decision, we compare the significance of our findings as a function of trajectory window by running our models 1 & 3 using output from trajectories with 1 day to 30-day window lengths.

## Results

**Fig. 1:**
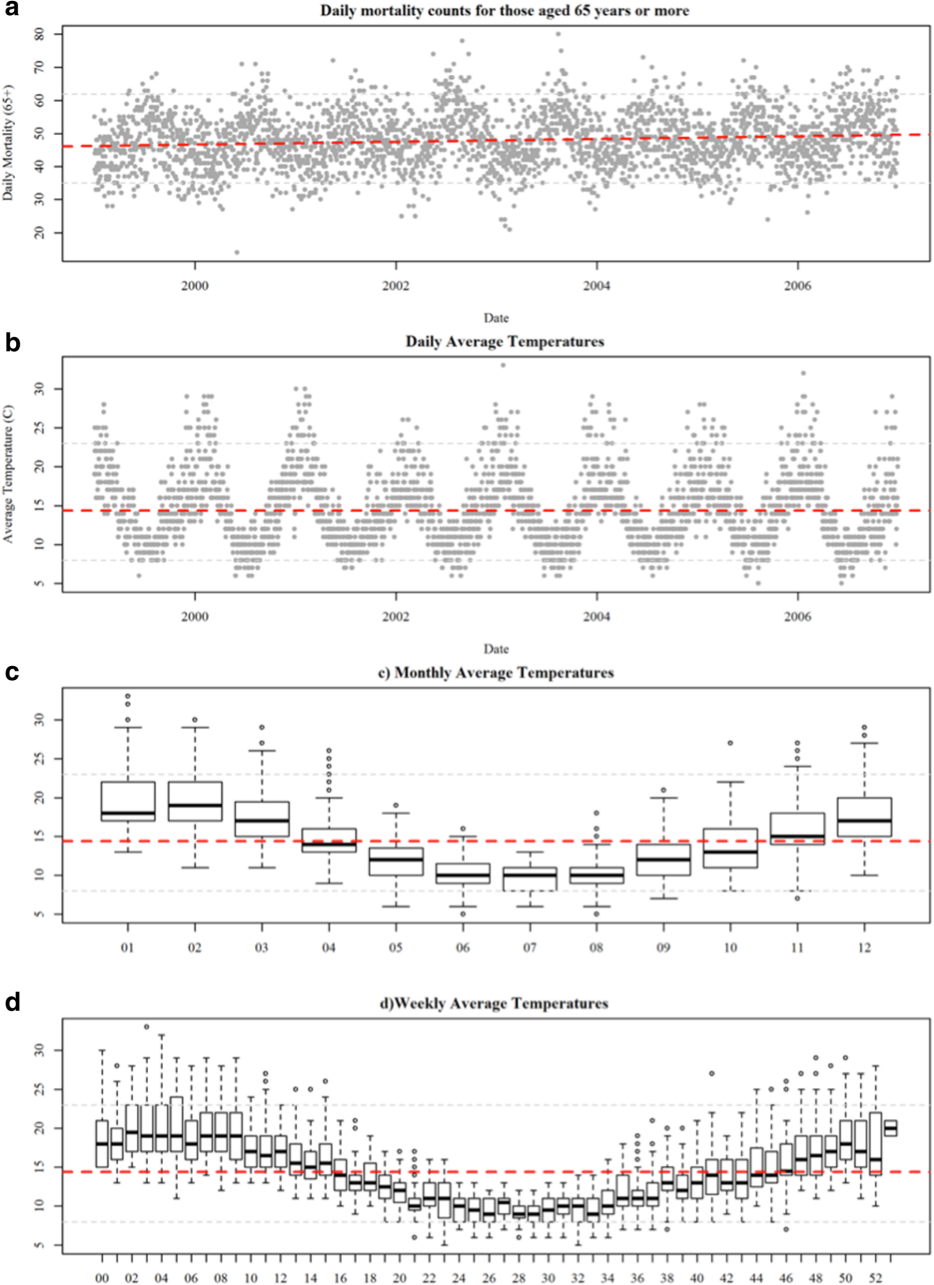
Panel **a** time-series plot of daily mortality; Panel **b** time-series of average temperature; Panel **c** boxplot of daily temperatures by month; Panel **d** boxplots of daily temperature by week of year. Note: *Light grey dashed lines* represent the 5^th^ and 95^th^ percentiles

The average number of all-cause-non-accidental daily deaths in the elderly population for Melbourne during the study period was 48 persons per day with a minimum of 14 and a maximum of 80 (Table 1). Mortality counts were higher in the cooler months but short-term fluctuations were obvious for all seasons (Fig. 1). A slight positive long-term trend was also visually apparent in the data and is consistent with trends in population growth [15].

**Table 1:** Summary statistics for elderly mortality (aged ≥ 65 years.) and meteorology in Melbourne, Australia 1999 to 2006

	Mean	SD	10^th^	25^th^	50^th^	75^th^	90^th^
Daily deaths ≥ (65 years)	47.9	8.2	38	42	47	53	58
Mean temperature (°C)	14.4	4.7	9	11	14	17	21
Dew-point temperature (°C)	7.8	3.5	4	5	7	10	12

During our study period, the mean daily average of temperature was 14 °C and the observed minimum daily average and maximum daily average temperatures were 5 and 33 °C, respectively. A strong oscillatory pattern was evident with peaks typically occurring during the warmer months of December through March (Fig. 1). In order to better understand shorter-term temperature behavior, we summarized daily average temperatures by month and week of the year during our study period. Monthly summaries reveal the greatest variability in daily temperature occurs during the warmer months (01–03; 1–12). Weekly summaries illustrate a similar pattern as variation was greater for weeks that occurred in the warmer months.

**Fig. 2:**
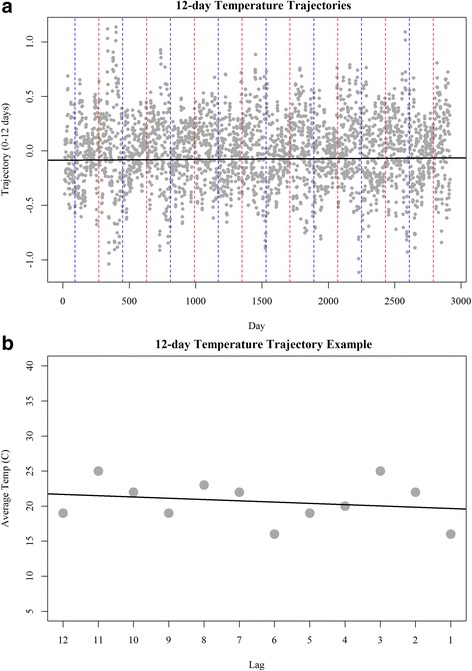
Panel **a** presents the slope of our 12-day temperature trajectories over time. Positive values indicate increasing temperatures, negative values indicate decreasing temperatures, and near zero indicate stability over the period of interest. Panel **b** provides an illustrative example of a 12-day trajectory

Trajectories were calculated for lengths of 1 to 30 days for average daily temperatures (Table 2). All trajectory windows were roughly normally distributed around 0 with variance reducing at similar rates as the trajectory window increases (Table 2). To illustrate, we chose to exemplify our method using a 12-day trajectory (Fig. 2). Evaluation of 12-day temperature trajectories illustrates no changes over the long-term; however, strong seasonal variations were evident, with larger magnitude trajectories being seen in the warmer months (Fig. 2a). This indicates that temperatures are more variable in warmer months. Using a 12-day period from our study, we illustrate how a trajectory captures the behavior of temperature change during our window of interest (Fig. 2b).

**Table 2:** Summary table of trajectory windows

Trajectory	Mean	SD	Median	Min	Max
0–1	−0.005	2.7	0.000	−12.0	9.0
0–2	−0.004	1.9	0.000	−8.0	7.5
0–3	−0.003	1.5	0.000	−5.6	5.7
0–4	−0.003	1.2	0.000	−4.3	4.3
0–5	−0.003	0.9	0.000	−3.3	3.8
0–6	−0.002	0.7	0.000	−2.4	3.5
0–7	−0.002	0.6	0.000	−2.0	2.7
0–8	−0.001	0.5	0.000	−1.7	2.3
0–9	−0.001	0.4	0.000	−1.6	1.8
0–10	−0.001	0.4	0.000	−1.5	1.6
0–11	−0.001	0.4	0.000	−1.3	1.3
0–12	−0.001	0.3	0.000	−1.1	1.1
0–13	−0.001	0.3	0.004	−0.9	1.1
0–14	−0.001	0.3	0.004	−0.8	1.0
0–15	−0.001	0.2	0.004	−0.8	0.9
0–16	−0.001	0.2	0.002	−0.7	0.9
0–17	−0.001	0.2	−0.001	−0.7	0.8
0–18	−0.001	0.2	−0.002	−0.7	0.8
0–19	−0.001	0.2	0.001	−0.6	0.7
0–20	−0.001	0.2	0.003	−0.6	0.6
0–21	−0.001	0.2	0.001	−0.6	0.5
0–22	−0.001	0.1	0.000	−0.5	0.5
0–23	−0.001	0.1	0.001	−0.5	0.5
0–24	−0.001	0.1	0.001	−0.5	0.5
0–25	−0.001	0.1	−0.002	−0.5	0.5
0–26	−0.001	0.1	−0.004	−0.4	0.5
0–27	−0.001	0.1	−0.005	−0.4	0.4
0–28	−0.001	0.1	−0.004	−0.4	0.4
0–29	−0.001	0.1	−0.005	−0.3	0.4
0–30	−0.001	0.1	−0.004	−0.3	0.3

**Fig. 3:**
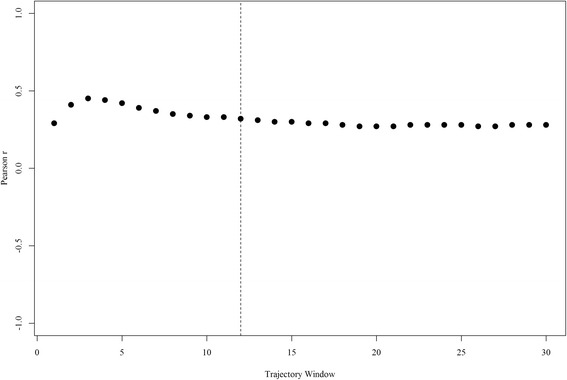
Pearson correlation between daily average temperature and temperature trajectories

The association between daily average temperature and the previous day’s temperature trajectory was weak over short windows and weakened as the trajectory window became larger (Fig. 3). Such low to moderate correlation indicates that multicollinearity should not be a major issue when applying this metric in a model with daily average temperature. It is important to note that correlations between trajectories were dependent upon their window differences, with similar windows being more correlated than dissimilar windows.

**Fig. 4:**
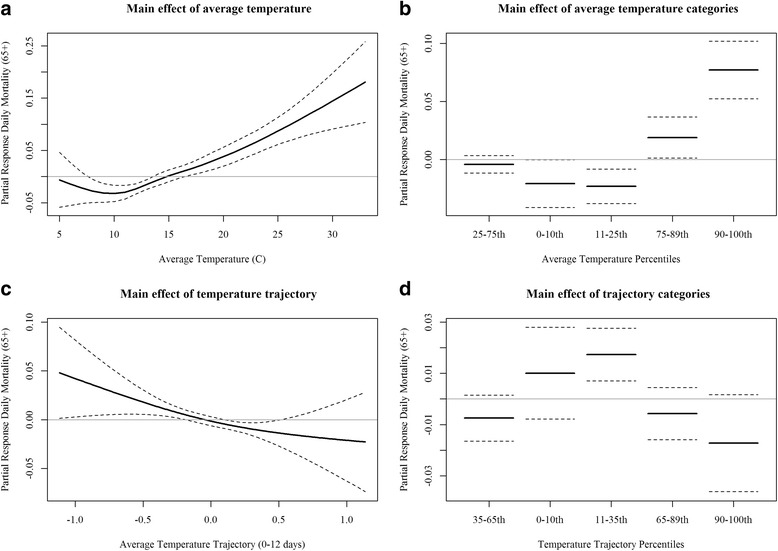
Main effects of average temperature and temperature trajectory (0–12 days) on mortality using a natural spline term in model 1 (Panel **ac**) and an indicator term in model 2 (Panel **bd**)

We investigated the main effects of same day average temperature and average temperature trajectory (0–12 days) using two models: **model 1** employs a natural spline term for our exposure metric of interest; and **model 2** employs an indicator term of percentile based categories. Both models identified temperature and temperature trajectory as being significantly associated with elderly mortality. In model 1, we see the ‘J’ shaped response curve for temperature showing that higher temperatures positively associate with elderly mortality in Melbourne (Fig. 4a). The effect of temperature maintained a similar response form in model 2, revealing an approximate 6 % increase in mortality on days when temperatures were above the 90^th^ percentile (≥21 °C) as compared to days in the referent 25-75^th^ percentile category (11–17 °C, Fig. 4b). For our temperature trajectory terms, models 1&2 suggest that periods of slightly decreasing temperatures over twelve days were most associated with daily mortality (Fig. 4 cd). In both models, the association for daily temperature was stronger than the association for temperature trajectory with mortality as indicated by residual deviance explained and *p*-values. For example, the residual deviance accounted for by temperature (*p* < 0.0001) in model 1 was 43.02 as compared to 10.08 for temperature trajectory (*p* = 0.0096).

Collectively, these results demonstrate that daily average temperature and temperature trajectory associate with elderly mortality in Melbourne. Although not presented here, it is important to note that evaluating daily maximum and minimum temperatures revealed similar findings as did the investigation of various lag terms (up to 14 days). The findings for average temperature were the strongest and thus were chosen to better facilitate testing for complex interactions. Sensitivity analysis of temperature trajectory window is presented in a later section.

**Fig. 5:**
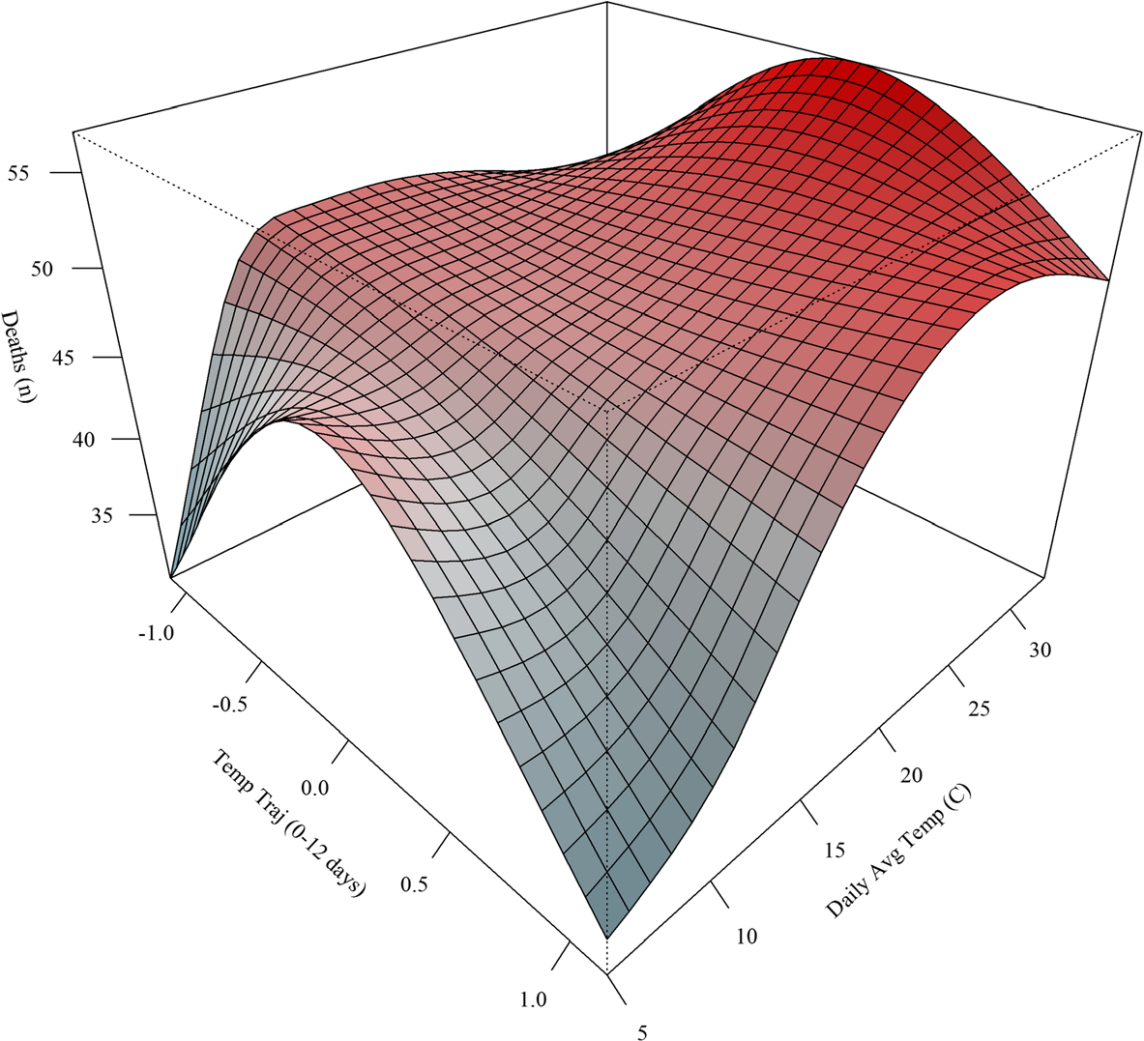
Product-term effects of average temperature and temperature trajectory (0–12 days) on mortality using a natural spline product-term in model 3

Results from a product term model, **model 3**, identified significant associations between average temperature (*p* < 0.0001), 12-day temperature trajectory (*p* = 0.01), and a product-term (*p* = 0.01). Visualization of the product-term effect demonstrates that the effect of daily average temperature varies between temperature trajectories (Fig. 5). We see that the effects peak when daily average temperatures are high during conditions when trajectories are near zero (i.e., periods of near stability). We also see mortality increases under higher temperatures with increasing trajectories. Daily mortality counts were found to be the lowest when temperatures were lowest and trajectories were at either extreme.

**Fig. 6:**
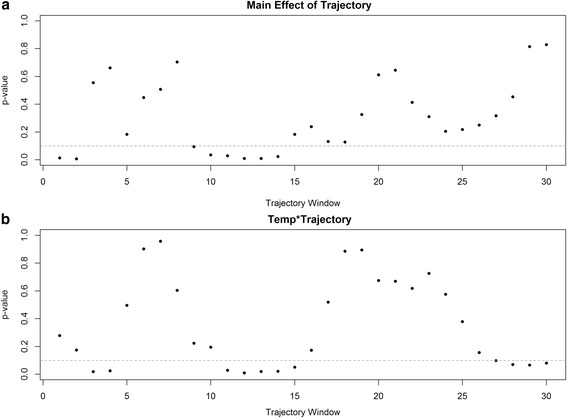
Panel **a** Model estimated *p*-values for natural spline main effects of temperature trajectories on mortality. Panel **b** Product-term *p*-values for temperature- temperature trajectory effects on mortality using a natural spline product-term. Note: *p*-values estimated using chi square test computed for analysis of deviance and *gray line* is at 0.1

Plotting *p*-value estimates for variable trajectory windows using our main effects (model 1) and product-term model (model 3) reveal that specification of window length is an important decision (Fig. 6). Examination of *p*-values as a function of trajectory window length revealed that main effects for our trajectory window were significant for very short windows (0–2 days), moderate windows (9–14 days), and long windows (24–30 days). For product-terms, windows of 3 to 4 days, 11 to 15 days, and 28 to 30 days were significant (under 0.1).

## Discussion

In this study, we sought to examine how the behavior of preceding days’ temperature impacted the relationship between daily temperature and elderly mortality. We achieved this by characterizing the rates of temperature changes on days preceding a daily temperature (i.e., temperature trajectory) and applied such characterization as an ‘exposure’ term in an epidemiologic model. As such, we were able to examine if the relationship between daily temperature and mortality varied by the preceding days trajectory.

Our study found a positive association between daily average temperature and elderly mortality (Fig. 5), with results demonstrating a ‘J’ shaped response as days characterized by higher temperatures were associated with the largest increases in mortality. These findings are consistent with other studies of Melbourne [4] and serve to strengthen understanding of the acute effects of heat on population health. In addition, we explored the influence of how daily temperatures progressed on days preceding an event using temperature trajectories on mortality as a main effect and as an effect modifier of daily temperature. Analyses of a main effect revealed a slight negative association between our trajectory metric and mortality that suggests near stability to slight decreasing temperature trend over the preceding twelve days increases mortality risk after accounting for other variables in our model. One explanation, for this result, is that our metric is identifying a ‘delayed’ effect in the data as periods shortly after temperature peaks could see increased mortality. Another explanation is that this cooling trend could be identifying impacts during the colder months; however, we are using daily mortality data so our results are skewed towards warm season and high temperature relationships.

Of course, our primary interest here was exploring the influence temperature trajectory has as an effect modifier rather than a main effect; nevertheless, further research into this relationship is warranted. Results from our product-term model revealed that the effect of daily average temperature was modified by the nature of the preceding days’ temperature trajectory (Fig. 6). This is the key finding of the study as it illustrates how the behavior of temperature on days leading up to a daily temperature event influences the association with mortality. We found that the highest temperatures in combination with relative stability over the preceding 12 days (i.e., trajectory near zero) corresponded with the peaks in daily mortality. This finding suggests that a ‘heat wave’ effect is occurring in Melbourne. This impact of temperature behavior agrees well with studies of heat/cold events in the United States as well as other locations around the globe [1, 16].

As our method is new, an important point of discussion is how it compares with previous approaches such as using more traditional lag terms and moving averages. The major distinction of our method is that it quantifies behavior change, in terms of directionality and magnitude, rather than quantifying behavior. This has several advantages. Interpretatively, health investigators can now explore the magnitude and direction of temperature behavior, a feature that improves understanding of the role of temperature variability on public health. Statistically speaking, when comparing trajectory of window (n) to lag-n term models, where *n* > 1, our approach is less sensitive to outlying days. Since the approach used to obtain trajectory values does not require the estimated line pass through the last value (lag-0) or require the first value (lag-n) to be the intercept, it estimates the overall temperature behavior. Furthermore, when comparing a trajectory of window (n) to a (*n* + 1)-day moving average, where *n* > 1, our approach is more robust in providing direction of change rather than simply quantifying behavior. Another benefit of our approach is that temperature trajectories were generally found to have little correlation with daily temperatures, a situation that provides the unique opportunity to approach modeling environmental effects on health outcomes with more detail without as much concern for issues of multicollinearity as may be found in lag term or moving average models.

Though our method is an innovative alternative to commonly used summary variables for temperature behavior, it does have limitations. One is that we performed a two-step approach and did not incorporate the precision of the trajectory estimates into our analysis; thus we have introduced uncertainties that make it harder to interpret confidence intervals. Considerations were made for weighting by the inverse standard error or using a coefficient of variation but neither was used as these methods need to be refined to improve interpretation. For example, weighting trajectories by the inverse standard error would inevitably produce values where the temperature behavioral characteristics would no longer be distinguishable because all days of near equal inversely proportional values of trajectory and its standard error would be near equal regardless of the trajectory value. Moreover, both approaches would deteriorate at trajectories close to zero as weighting by the inverse standard error would only produce another value close to zero, regardless of the standard error, and the coefficient of variation would approach infinity. Generally speaking, the expected impact of this uncertainty is somewhat analogous to exposure misclassification and thus bias towards the null is the likely result. Further work should be done to include the precision of the trajectory estimate. Another limitation of this work is that our models treat the trajectory of temperature as linear, an assumption that may not always be true. As such, our analysis may have missed more subtle temperature behavior impacts on health. In addition to methodological limitations, there are also additional limitations to the interpretability of our findings. For example, our study focuses on a single city and thus it is possible that our results may not be found in other locations. Thus, to improve the generalizability of our findings, future multi-city studies are recommended.

Possible future directions are rich with opportunities as there are several alternative approaches that could be used to capture patterns in changing temperatures over time. One such possibility could be considering a ‘floating-window’ where the size of the trajectory window is a function of standardized expected temperature. Another alternative would be to include some function of temperatures in the models that would reduce concerns about uncertainty. However, such approaches need to be more fully developed as models are complex. Additionally, possibilities include modeling a mixture of trajectories in the context of single predictor models (i.e., temperature, single-pollutant, etc.) and multi-predictor models (i.e., multi-pollutant) models.

## Conclusion

There is abundant interest in potential health effects attributed to climate. This study, through its development and application of a novel temperature behavior summary variable, has led to a measure that captures the behavior of temperature leading up to present day- enhancing our ability to interpret the association of temperature and mortality. Overall, we found temperature trajectories are a very useful tool to investigate the association of temperature and mortality. Finally, our methodology provides a new tool for public health scientists to better understand and prepare for the health impacts associated with a changing climate.

## Abbreviations

DF: Degrees of freedom
GLM: Generalized linear model
